# Paradoxes of Elimination and Persistence: Neglected Tropical Diseases in Ecuador Compared to Latin America and the Caribbean

**DOI:** 10.1155/jotm/2977701

**Published:** 2026-09-20

**Authors:** José Daniel Sánchez Redrobán

**Affiliations:** ^1^ Facultad de Ciencias de la Salud y Bienestar Humano, Universidad Tecnológica Indoamérica, Quito, Ecuador

**Keywords:** Chagas disease, dengue, Ecuador, Latin America, leishmaniasis, neglected tropical diseases, WASH

## Abstract

**Objective:**

To describe, from a comparative Latin American perspective, the progress and gaps in the elimination of neglected tropical diseases (NTDs) in Ecuador, integrating recent data on arboviruses, American trypanosomiasis, leishmaniasis, and structural determinants such as water, sanitation, and hygiene (WASH).

**Methods:**

A narrative review with a descriptive comparison of secondary data based on secondary sources published between 2015 and 2026. Sources were identified through structured searches of PubMed/MEDLINE, the PAHO Institutional Repository for Information Sharing (IRIS), the WHO Global Health Library, and the WHO/UNICEF Joint Monitoring Programme (JMP) data portal, supplemented by targeted retrieval of official technical reports from WHO, PAHO, UNICEF, and Ecuador’s Ministry of Public Health. Comparative indicators were constructed between Ecuador and the Region of the Americas or Latin America and the Caribbean (LAC) for: (a) burden and elimination of selected NTDs; (b) recent dengue dynamics; and (c) WASH service coverage. A synthetic two‐panel table with key indicators was prepared, and an interpretive narrative analysis was conducted.

**Results:**

Globally, NTD and malaria prevalence decreased 58% between 1990 and 2021, but the reduction in disability‐adjusted life years was only 18%, with a disproportionate burden still in low‐ and middle‐income regions. In the Americas, Ecuador was the second country to certify onchocerciasis elimination in 2014, following intensive mass drug administration (MDA) campaigns with ivermectin, with posttreatment surveillance confirming absence of transmission. The Region recorded a record dengue outbreak in 2024 with over 12.6 million suspected cases and more than 7700 deaths, concentrated in four large Latin American countries; through epidemiological Week 21 of 2025, more than 3 million cases had been reported, of which 23,623 corresponded to Ecuador. In 2020, 75% of the LAC population used safely managed drinking water and 34% used safely managed sanitation; nevertheless, 17 million people still lacked access to even basic water services and 72 million lacked basic sanitation. In Ecuador, basic drinking water‐service coverage reached 95.7% in 2022, but only 41.6% used safely managed sanitation, and in rural areas, substantial proportions of unimproved water sources, surface water use, and open defecation persist. According to GBD 2023 data, the age‐standardized DALY rate for NTDs and malaria combined was 193.8 per 100,000 in Ecuador (95% UI: 139.4–267.6), substantially lower than Bolivia (388.9) and Brazil (313.3).

**Conclusions:**

Ecuador illustrates a regional paradox that is primarily institutional and programmatic rather than biological. Vertical programs endowed with a dedicated external initiative, a donated single‐dose drug, a verifiable elimination endpoint, and long‐term technical accompaniment—as in onchocerciasis—can interrupt transmission of a historical NTD, whereas the persistence of structural determinants—poverty, precarious urbanization, and WASH gaps—sustains the burden of other NTDs and arboviruses. PAHO’s Communicable Disease Elimination Initiative offers an opportunity to articulate elimination goals with rural water and sanitation policies, entomological surveillance, and intercultural territorial approaches. Sustained investments in WASH, integrated surveillance, and strengthened primary care are required to transform punctual elimination achievements into structural health equity gains.

## 1. Introduction

Neglected tropical diseases (NTDs) and other poverty‐linked communicable diseases continue to affect more than one billion people worldwide, despite progress made in recent decades [1, 2]. A recent analysis from the Global Burden of Disease study showed that between 1990 and 2021, the combined prevalence of NTDs and malaria decreased 58%, while the burden in disability‐adjusted life years (DALYs) decreased only 18%, demonstrating that residual morbidity is concentrated in historically marginalized populations [3]. Latin America is paradigmatic: While harboring emblematic foci of elimination—such as onchocerciasis—it concentrates most of the world’s population infected with *Trypanosoma cruzi* and a growing burden of urban arboviruses.

Chagas disease, caused by *T. cruzi*, affects more than 7 million people worldwide, most residing in or from Latin America, where it remains intimately linked to precarious housing conditions and barriers to timely diagnosis and treatment [2, 4]. Cutaneous and visceral leishmaniasis remain a public health problem in several countries of the Region, with heterogeneous trends; recent PAHO reports describe an increase in cutaneous leishmaniasis cases in countries such as Argentina, Costa Rica, Ecuador, Mexico, and Suriname, despite a global reduction in cases [5]. Simultaneously, soil‐transmitted helminthiases (STH) and schistosomiasis remain associated with environments with insufficient access to safe water and adequate sanitation, highlighting the critical role of water, sanitation, and hygiene (WASH) interventions in the elimination agenda [1, 6].

The coexistence of NTD elimination achievements and persistent disease burden is not a new phenomenon. Hotez et al. documented that Latin America’s NTD landscape has long been defined by this paradox: Targeted vertical programs can reduce or interrupt specific transmission foci, while the structural conditions—poverty, inadequate housing, WASH deficits—that sustain multidisease vulnerability remain largely unchanged [7]. Engels and Zhou subsequently argued that durable NTD control requires integration with primary healthcare and poverty alleviation strategies, not pharmacological scale‐up alone [8]. Ecuador’s experience contributes to this body of evidence not as an isolated curiosity, but as a rigorously documented contemporary instance: a middle‐income country that has achieved WHO certification of elimination for one NTD while simultaneously facing a record arboviral outbreak, and while Chagas and leishmaniasis burdens remain unresolved—all in the context of a dual WASH profile characterized by high basic coverage but persistent safely managed deficits.

### 1.1. Defining the Paradox: Three Candidate Mechanisms and One Dominant Explanation

Because the term “paradox” can be used loosely, we state explicitly what is, and what is not, paradoxical about Ecuador’s divergent NTD outcomes. Three mechanisms could in principle explain the contrast between certified onchocerciasis elimination and the persistence of Chagas disease, STH, leishmaniasis, and dengue.

*A biological and ecological mechanism.* Onchocerciasis has no nonhuman reservoir, a single‐competent vector genus with restricted breeding ecology, and a parasite whose reproductive output can be suppressed below the transmission threshold by repeated ivermectin dosing. *Trypanosoma cruzi* has more than 180 mammalian reservoir species and sylvatic triatomine populations that cannot be eradicated; STH transmission is sustained by an environmental egg reservoir insensitive to chemotherapy; *Aedes aegypti* breeds in domestic containers throughout the urban matrix [7, 9, 10].

*An institutional and programmatic mechanism.* Onchocerciasis control in the Americas was delivered by a dedicated regional initiative—the Onchocerciasis Elimination Program for the Americas (OEPA), launched in 1993 as an international partnership with sustained external financing—using a single donated drug (ivermectin, through the Mectizan Donation Program), a simple biannual delivery model with an explicit coverage target of at least 85% of the eligible population, and an internationally codified endpoint with agreed criteria for stopping mass drug administration (MDA) and verifying interruption of transmission [11–13]. None of the other NTDs discussed here has had an equivalent architecture: Benznidazole and nifurtimox require prolonged, poorly tolerated courses rather than single‐dose community distribution; STH preventive chemotherapy (PCT) has no verifiable transmission‐interruption endpoint; and dengue has no drug‐based interruption strategy at all.

*A political-economic mechanism.* The decisive levers for the persistent NTDs lie outside the health ministry’s budget and authority—housing quality for Chagas disease, rural sanitation for STH, continuous piped water supply, and solid waste management for dengue—and therefore lack the short political payback and the auditable “finish line” that made onchocerciasis elimination institutionally attractive [8, 14].

We argue that the biological mechanism is necessary but not sufficient, and that the dominant explanation is institutional and programmatic. Geographical restriction and clinical visibility do not by themselves generate two decades of ≥ 85% MDA coverage: external financing, predictable drug supply, a single simple intervention, and a measurable endpoint do. Table 1 contrasts these programmatic attributes across the four disease groups analyzed. Framed in this way, the paradox is diagnostic rather than rhetorical: Ecuador demonstrated world‐class programmatic capability under conditions of external financing, technical accompaniment, and a verifiable endpoint, and that capability has not transferred to diseases whose control requires domestically financed, cross‐sectoral, open‐ended investment. The paradox therefore identifies both a transferable asset—community‐based distribution and surveillance capacity—and a nontransferable one: the vertical funding‐and‐endpoint architecture itself.

**TABLE 1:** Programmatic architecture of onchocerciasis elimination compared with persistent NTDs and arboviruses in Ecuador and Latin America.

Attribute	Onchocerciasis	STH	Chagas disease	Dengue
Dedicated regional initiative with external financing	Yes (OEPA, 1993–)	No (integrated into national PCT)	Subregional intergovernmental initiatives, limited external financing	No dedicated elimination financing
Donated medicine	Yes (Mectizan Donation Program)	Albendazole/mebendazole donations, variable		Not applicable
Intervention complexity	Single annual/biannual oral dose	Single oral dose, repeated indefinitely	60‐day course, tolerability and monitoring requirements	Multicomponent vector management
Verifiable elimination endpoint	Yes (WHO criteria for stopping MDA and verifying interruption)	No transmission–interruption endpoint	Interruption of domiciliary vector transmission only	None defined
Principal transmission interface	Vector, focal, and riverine	Environmental (fecal contamination of soil)	Domestic vector plus sylvatic reservoirs, congenital and oral routes	Domestic and peridomestic containers
Decisive sector	Health	Water and sanitation	Housing and social protection	Water supply, solid waste, urban planning
Dependence on WASH or housing investment	Negligible	Determinant	Determinant (housing quality)	Determinant (supply continuity, storage, waste)

*Note:* OEPA: Onchocerciasis Elimination Program for the Americas; WASH: water, sanitation, and hygiene. Compiled by the author from [6, 9–13, 15, 16].

Abbreviations: MDA = mass drug administration, PCT = preventive chemotherapy, STH = soil‐transmitted helminthiases.

Although dengue is not uniformly classified as an NTD within all global health frameworks, its inclusion in this analysis is conceptually warranted. PAHO’s Communicable Disease Elimination Initiative recognizes dengue among priority communicable diseases [17], and a growing body of literature demonstrates that its transmission is governed by the same structural determinants—precarious housing, WASH gaps, poverty, and inequitable access to healthcare—that define “neglect” [8, 18]. Ackley et al. confirm that successful NTD interventions require multisectoral action on these determinants, a framework equally applicable to arboviral diseases [14]. Treating dengue and other arboviruses as part of the NTD discussion therefore reflects analytical coherence rather than taxonomic imprecision.

In contrast with this persistence, the Region of the Americas has achieved important elimination milestones. PAHO’s regional Communicable Disease Elimination Initiative aims to eliminate more than 30 diseases and related conditions by 2030 [17]. An emblematic example is onchocerciasis elimination: Colombia, Ecuador, Mexico, and Guatemala achieved WHO certification between 2013 and 2016, after decades of mass ivermectin administration programs [19, 20]. In Ecuador, the Esmeraldas focus went from being one of the most intensely hyperendemic areas in the Americas to a scenario free of transmission verified through entomological and serological surveillance [19].

However, these achievements coexist with a scenario of epidemic reemergence of other diseases. In 2024, the Region of the Americas registered more than 12.6 million suspected dengue cases and more than 7700 deaths—the highest number on record—with approximately 90% of cases concentrated in Brazil, Argentina, Colombia, and Mexico [21]. In 2025, through epidemiological Week 21, PAHO reported more than 3 million cases, including more than 1.3 million confirmed cases and 1324 deaths in the Region [9].

The structural determinants of these paradoxes are evident. The WHO/UNICEF Joint Monitoring Programme (JMP) for WASH estimated that in 2020, three‐quarters of the Latin American and Caribbean (LAC) population used safely managed drinking water and one‐third had access to safely managed sanitation; however, 17 million people still lacked basic water services and 72 million lacked basic sanitation, with marked urban–rural gaps [22]. In Ecuador, national indicators show high coverage of basic drinking water services (95.7% in 2022), but more modest coverage of safely managed sanitation (41.6%), along with important territorial disparities [23]. A report by Ecuador’s Ministry of Environment, Water and Ecological Transition (MAATE) and UNICEF documented that in rural areas, a substantial proportion of the population still uses unimproved or surface water sources, and open defecation practices persist, with significant economic and health costs [24].

Ecuador, therefore, illustrates the paradoxes of elimination and persistence: certified elimination of a historical NTD, coexisting with a significant burden of arboviruses, Chagas disease, leishmaniasis, and helminthiases, fueled by unresolved structural determinants. The objective of this work is to situate the Ecuadorian experience in the recent Latin American context, integrating quantitative evidence and regional elimination frameworks, with emphasis on the role of WASH and social determinants of health. This analysis is framed explicitly as a policy‐relevant synthesis, drawing on secondary institutional data, rather than a primary analytical study [25].

## 2. Materials and Methods

### 2.1. Study Design

A narrative review with descriptive comparison of secondary data was conducted. The analysis focused on comparing aggregate indicators of NTDs and selected structural determinants between Ecuador and LAC or the Region of the Americas, depending on the source’s geographic denominator.

### 2.2. Literature Search and Source Selection

Sources were identified through a structured search of the following databases and repositories: PubMed/MEDLINE, the PAHO Institutional Repository for Information Sharing (IRIS), the WHO Global Health Library, and the WHO/UNICEF JMP data portal. Searches were conducted between January and March 2026 and covered materials published from January 2015 through March 2026, with the exception of foundational references (e.g., methodological or theoretical works) published prior to this window. Search terms included combinations of: “neglected tropical diseases,” “NTD,” “elimination,” “Ecuador,” “Latin America,” “WASH,” “water sanitation hygiene,” “dengue,” “arboviruses,” “Chagas disease,” “leishmaniasis,” “onchocerciasis,” “mass drug administration,” “preventive chemotherapy,” and “social determinants.”

Documents were included if they: (a) provided epidemiological data or programmatic evaluations relevant to NTDs in Ecuador or the LAC region; (b) reported WASH coverage indicators at national or regional level; (c) described elimination frameworks or policy syntheses from WHO, PAHO, or comparable institutions; or (d) offered methodological or theoretical perspectives directly applicable to the analytical framework. Gray literature was included when it constituted the authoritative primary source for an indicator (e.g., JMP reports, national SDG profiles, WHO certification documents). Journalistic sources were excluded as primary evidence sources. Where multiple sources reported the same indicator with differing values or reference years, the most recent primary institutional source was preferred and any discrepancy was noted in the text or table footnotes.

**FIGURE 1:**
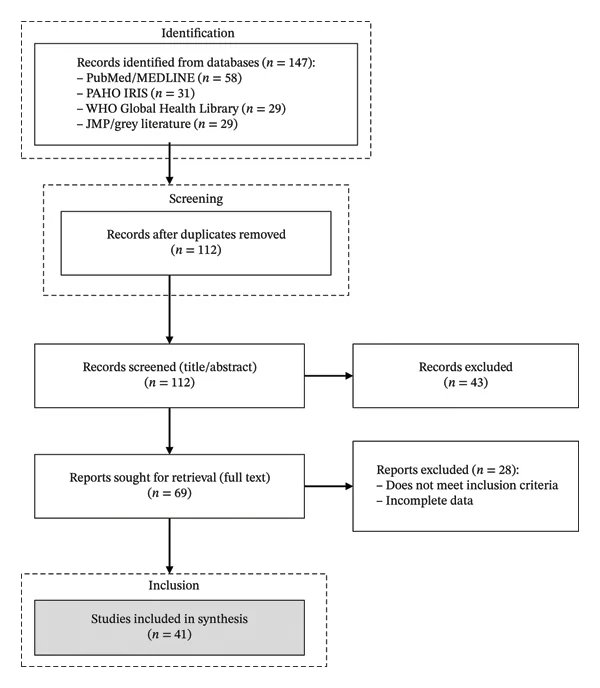
PRISMA 2020 flow diagram documenting literature search and document selection process. A total of 147 records were initially identified, of which 41 met inclusion criteria for data extraction and narrative synthesis. Exclusions at the screening stage were primarily journalistic sources, duplicate reports, or thematically unrelated content; exclusions at full‐text review were due to lack of Ecuador‐specific data, insufficient methodological detail, or redundancy with more authoritative sources.

A total of 147 records were initially identified through database searches: 58 from PubMed/MEDLINE, 31 from PAHO IRIS, 29 from WHO Global Health Library, and 29 from JMP and other gray literature sources. After deduplication, 112 records underwent title and abstract screening, of which 43 were excluded as not meeting inclusion criteria (primarily journalistic sources, duplicate reports, or thematically unrelated content). The remaining 69 reports underwent full‐text review; 28 were excluded due to lack of Ecuador‐specific data, insufficient methodological detail, or redundancy with more authoritative sources. A final set of 41 documents was retained for data extraction and synthesis. Figure 1 presents the PRISMA flow diagram documenting this selection process.

### 2.3. Information Sources

The following secondary sources were used:

• PAHO reports and databases, including the Communicable Disease Elimination Initiative [17], epidemiological reports on leishmaniasis in the Americas [5], and dengue situation reports for 2024 and for epidemiological Week 21 of 2025 [9, 21].
• WHO documents on onchocerciasis elimination in the Americas [20], reports on schistosomiasis and STHs [1], the WHO NTD Roadmap 2021–2030 [6], and the Global Report on Neglected Tropical Diseases 2025 [26].
• Indexed scientific articles on onchocerciasis elimination in Ecuador [19], global and regional burden of NTDs [3], geodemography of Chagas disease in Ecuador [27], Chagas disease clinical overview [4], NTD intervention frameworks [7, 8, 14, 25], and arbovirus dynamics in Latin America [16, 18].
• Reports from the WHO/UNICEF JMP for WASH, including the regional summary for LAC [22] and the global update 2000–2022 [28].
• SDG indicator data for Ecuador obtained from United Nations country profiles and World Bank series [23, 29].
• The MAATE–UNICEF report on costs associated with inadequate WASH services in rural Ecuador [24].
• Global Burden of Disease 2023 data from the Institute for Health Metrics and Evaluation (IHME), accessed via GBD Results Tool [30].

### 2.4. Variables and Indicators

For NTDs and arboviruses, the following indicators were extracted when available:

• Cumulative suspected and confirmed dengue cases and deaths for the Region of the Americas (2024 year‐end; 2025 through EW 21), and for Ecuador (2025 through EW 21)[9, 21].
• Number of countries with onchocerciasis elimination certification in the Americas [19, 20].
• Cumulative number of confirmed severe Chagas disease cases in Ecuador (2013–2019) and geographic distribution [27].
• Trend in cutaneous leishmaniasis incidence for the LAC region and Ecuador [5].
• Age‐standardized DALY rates for NTDs and malaria (GBD Cause 344) from GBD 2023 [30].

For WASH and structural determinants:

• Proportion of population using safely managed and at least basic drinking water services (SDG 6.1 definition), for LAC and Ecuador separately [22, 23, 28].
• Proportion of population using safely managed and at least basic sanitation services (SDG 6.2), for LAC and Ecuador [22, 23].
• Water and sanitation coverage in rural Ecuador, disaggregated by service category (unimproved, surface water, open defecation) [24].

### 2.5. Selection of Comparator Countries

Bolivia, Brazil, Colombia, and Peru were selected a priori as comparators for the burden analysis using five explicit criteria. First, ecological and epidemiological comparability: all four are South American countries sharing Andean and/or Amazonian ecological zones with Ecuador and are co‐endemic for the same NTD complex (Chagas disease, cutaneous leishmaniasis, STH, and dengue). Second, contrasting programmatic trajectories that make the comparison analytically informative: Colombia was the first country worldwide to obtain WHO verification of onchocerciasis elimination (2013), immediately preceding Ecuador [12, 31]; Brazil is the only country in the Region with endemic schistosomiasis and carries the largest absolute dengue burden [21, 32]; Bolivia has historically reported the highest *T. cruzi* seroprevalence in the Americas [9, 15]; and Peru provides an Andean comparator with a sanitation profile close to Ecuador’s.

**FIGURE 2:**
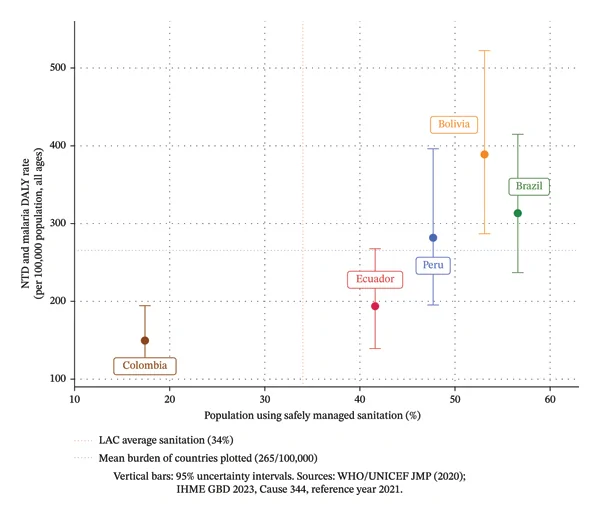
Relationship between safely managed sanitation coverage and NTD + malaria burden in selected South American countries, 2020–2021. Ecuador (highlighted in red) demonstrates an intermediate position: higher sanitation coverage than Colombia and comparable to regional averages, yet with a substantial disease burden (193.8 DALYs/100,000). Vertical bars denote 95% uncertainty intervals; the dotted reference lines mark the LAC average for safely managed sanitation (34%), and the unweighted mean burden of the five countries plotted (265 per 100,000). No regression line is fitted: with five observations, overlapping uncertainty intervals for Ecuador, Colombia, and Peru, and a cause group that aggregates non–WASH‐mediated malaria, the plot is descriptive only and causal inference at the individual level is not warranted (ecological fallacy). Source: Sanitation data from WHO/UNICEF JMP (2020); burden data from GBD 2023 (IHME, 2024), Cause 344, both sexes, all ages, reference Year 2021 (Supporting File S1).

Third, availability of GBD 2023 country‐level estimates for Cause 344 with comparable uncertainty intervals. Fourth, availability of matching WHO/UNICEF JMP national estimates for the same period, permitting the ecological juxtaposition presented in Figure 2. Fifth, income‐group comparability, since all five countries are classified as middle‐income, avoids contrasting Ecuador with structurally different high‐ or low‐income settings.

Mexico and Guatemala—the two remaining countries verified free of onchocerciasis in the Americas—were deliberately excluded because their Mesoamerican disease ecology, vector species composition, and health‐system organization differ substantially from the Andean‐Amazonian setting. Venezuela was excluded because the interruption of routine surveillance reporting and the persistence of the binational Yanomami onchocerciasis focus render its national estimates noncomparable for this purpose [12]. Small Caribbean states were excluded because low case counts produce unstable age‐standardized rates.

### 2.6. Sources Added During Peer Review

In response to reviewer requests for greater specification of disease‐level control strategies, of the WASH mechanisms operating in each transmission cycle, and of the programmatic architecture underlying the onchocerciasis result, a limited number of additional sources were retrieved through citation chaining from the documents already included. Because these were not identified through the original database searches, they are not incorporated into the PRISMA counts reported above or in Figure 1, which describe the systematic component of the search only.

### 2.7. Analysis

A comparative descriptive analysis was performed. Regional LAC indicators were extracted directly from PAHO and JMP reports, while national data for Ecuador were extracted from SDG country profiles, World Bank Development Indicators, and the MAATE–UNICEF report. GBD 2023 data were extracted from the IHME GBD Results Tool, focusing on Metric 3 (rate per 100,000 population) for Cause 344 (NTDs and malaria). The complete extraction returned by the Results Tool—the five countries, the three metrics released by the tool (number of DALYs, percentage of total DALYs, and rate per 100,000 population) and their 95% uncertainty intervals—is deposited unmodified as Supporting File S1 and is the source of the burden estimates reported below. Data from different reporting years are presented together in Tables 2, 3, and 4 with year labels attached to each indicator to facilitate transparency. Where LAC and Ecuador data referred to different years, no direct arithmetic comparison was made; results are described narratively with appropriate qualification.

**TABLE 2:** DALY rate for neglected tropical diseases and malaria (per 100,000 population; both sexes, all ages), GBD 2023.

Country	DALY rate (per 100,000)	95% uncertainty interval
Bolivia	388.9	286.9–522.4
Brazil	313.3	237.0–414.8
Colombia	149.6	117.1–194.5
**Ecuador**	**193.8**	**139.4–267.6**
Peru	281.8	195.3–396.2

*Note:* Source: Global Burden of Disease Collaborative Network. Global Burden of Disease Study 2023 (GBD 2023) Results. Institute for Health Metrics and Evaluation (IHME), 2024. DALY: disability‐adjusted life year. Data correspond to cause 344 (neglected tropical diseases and malaria), both sexes, all ages, rate per 100,000 population, reference Year 2021. The complete extraction underlying this table, including the numerical and percentage metrics and the citation generated at download, is provided as Supporting File S1. Bold values highlight the data corresponding to Ecuador, the focal country of this study.

**TABLE 3:** WASH coverage indicators: Latin America and the Caribbean (LAC) vs. Ecuador.

Indicator	LAC	Ecuador
*Basic and safely managed drinking water services*		
Population using safely managed drinking water (%)	75 (2020)^a^	n.r.1
Population using at least basic drinking water services (%)	96 (2020)^a^	95.7 (2022)^b^
Population lacking basic drinking water service (millions)	17 (2020)^a^	

*Basic and safely managed sanitation services*		
Population using safely managed sanitation (%)	34 (2020)^a^	41.6 (2022)^b^
Population using at least basic sanitation services (%)	89 (2020)^a^	—
Population lacking basic sanitation services (millions)	72 (2020)^a^	—
Open defecation (% of population)	< 1 (2020)^a^	≈3, rural (2020)^c^

*Rural WASH indicators (Ecuador only)*		
Rural population using unimproved water sources (%)	—	7 (2020)^c^
Rural population using surface water (%)	—	6 (2020)^c^
Rural population using limited or unimproved sanitation (%)	—	9 (2020)^c^

*Note:* Sources: —: not available or not applicable at this level of aggregation for this geographic unit.

^a^WHO/UNICEF JMP LAC regional summary 2000–2020 [22].

^b^Ecuador SDG country profile [23] and World Bank Development Indicators [29].

^c^MAATE–UNICEF rural Ecuador 2022 [24]. LAC: Latin America and the Caribbean.

**TABLE 4:** NTD and arbovirus disease burden indicators: Region of the Americas and Ecuador.

Indicator	Region of the Americas	Ecuador
*Onchocerciasis*		
Countries with WHO certification of elimination	4 (2013–2016)^d^	Certified 2014^d^

*Dengue (2024, year-end)*		
Suspected cases	> 12.6 million^e^	—
Deaths	> 7700^e^	—

*Dengue (2025, EW 1–21)*		
Suspected cases	3,035,646^f^	23,623^f^
Confirmed cases	1,210,296^f^	—
Severe dengue cases	3,830^f^	—
Deaths	1,324^f^	—

*Chagas disease (Ecuador, 2013–2019)*		
Confirmed severe (hospitalized) cases	—	439^g^
Provinces with reported cases	—	20 of 24^g^

*Leishmaniasis (most recent period)*		
Cutaneous leishmaniasis trend	Slight regional decrease^h^	Increasing trend^h^

*Note:* Sources: —: not available or not the appropriate unit of comparison.

Abbreviation: EW = epidemiological week.

^d^WHO WER 2014 [20] and Guevara et al. 2018 [19].

^e^PAHO end‐of‐year dengue report 2024 [21].

^f^PAHO EW21 dengue report 2025 [9].

^g^Velasquez‐Ortiz et al. [27] (severe hospitalized cases only; does not represent total national burden).

^h^PAHO Leishmaniasis Report No. 13, 2024 [5].

An interpretive narrative analysis was developed contextualizing results within the framework of PAHO’s Communicable Disease Elimination Initiative and the social determinants of health approach. Passages that reflect the authors’ analytical interpretation—rather than direct reporting from cited sources—are explicitly framed as such in the text.

As this study is exclusively based on aggregated secondary sources available in the public domain, approval from a research ethics committee was not required.

## 3. Results

### 3.1. Global and Regional Burden of NTDs

The 2021 Global Burden of Disease Study analysis showed that globally, the combined prevalence of NTDs and malaria decreased 58.1% between 1990 and 2021, while DALYs decreased 18.1% in the same period [3]. Nevertheless, NTDs remain responsible for an important fraction of disease burden in low‐ and middle‐income regions, with geographic concentration in sub‐Saharan Africa, Asia, and Latin America [3]. WHO’s 2025 Global NTD Report places the number of people requiring interventions against NTDs at over 1.6 billion globally [26].

In the Region of the Americas, PAHO has identified Chagas disease, leishmaniasis, schistosomiasis, STHs, onchocerciasis, fascioliasis, and echinococcosis, among others, as priorities within the Communicable Disease Elimination Initiative [17]. The region has achieved notable progress in interrupting vector transmission of diseases such as onchocerciasis, but important foci of Chagas disease, leishmaniasis, and helminthiases linked to structural inequities persist.

### 3.2. Onchocerciasis Elimination and Lessons for the Region

In September 2014, the WHO certified onchocerciasis elimination in Ecuador, following the recommendation of an international verification team that confirmed the absence of transmission in the Esmeraldas focus and its satellite foci [19, 20]. The national program, in coordination with the OEPA, implemented mass ivermectin administration (MDA) twice a year for nearly two decades, achieving coverage exceeding 85% of the target population, accompanied by entomological surveillance with *Simulium exiguum* capture and serological monitoring in children [19].

Ecuador thus became the second country in the Americas to obtain certification, after Colombia, and before Mexico and Guatemala [20]. This achievement demonstrates that even in hyperendemic foci with highly competent vectors, elimination is feasible through sustained MDA strategies, community participation, and intensive surveillance.

Regarding STH—another group amenable to MDA—Ecuador has implemented periodic PCT targeting school‐age children in endemic areas, consistent with WHO and PAHO recommendations [1, 6]. Unlike onchocerciasis, however, STH control through PCT does not interrupt transmission unless supported by concurrent improvements in WASH conditions, since reinfection from environmental contamination occurs rapidly in settings where fecal–oral pathways remain open [8, 14]. The replicability of the onchocerciasis model is therefore limited for STH and other NTDs with animal reservoirs, multiple vectors, or predominantly oral–fecal or domestic transmission routes.

### 3.3. Dengue and Arboviruses: Epidemic Record in the Americas

The contrast between vector‐targeted MDA success and arboviral persistence is stark. According to PAHO’s year‐end dengue epidemiological report for 2024, the Region of the Americas registered more than 12.6 million suspected dengue cases and more than 7700 deaths, representing the highest recorded annual toll since systematic surveillance began [21]. Brazil, Argentina, Colombia, and Mexico concentrated approximately 90% of cases and 88% of deaths, reflecting the weight of large, densely populated urban and periurban areas.

PAHO’s epidemiological update for Week 21 of 2025 reported 3,035,646 suspected dengue cases in the Region of the Americas, of which 1,210,296 (40%) were confirmed, 3830 (0.1%) classified as severe, and 1324 deaths (case fatality rate of 0.044%) [9]. Twenty countries reported cases at that cutoff; Brazil contributed more than 2.7 million, while Ecuador reported 23,623 accumulated cases with 1212 cases in epidemiological Week 21 [9].

In Ecuador, the available epidemiological data are consistent with patterns documented in the regional literature: growing arboviral cycles associated with climatic variability, accelerated urban expansion, and deficiencies in solid waste management and vector control in periurban settings [16, 18]. The cocirculation of dengue, Zika, and chikungunya viruses creates a complex epidemiological scenario. These observations are consistent with the eco‐bio‐social determinants of arboviral transmission described across Latin America [14], though direct causal attribution for Ecuador‐specific dengue trends would require dedicated epidemiological studies beyond the scope of this secondary analysis.

### 3.4. Chagas Disease and Leishmaniasis in Ecuador

Although Chagas disease has lost media visibility, it continues to be a high‐impact NTD. The WHO 2025 Global NTD Report estimates that approximately 6–7 million people worldwide are currently infected with *T. cruzi*, the vast majority in Latin America, though migration has internationalized the burden to nonendemic countries [4, 26]. A geodemographic study of *severe* hospitalized Chagas cases in Ecuador between 2013 and 2019 identified 439 confirmed cases, with a predominance of chronic forms (75.4%) and a distribution encompassing 20 of 24 provinces, mainly El Oro, Guayas, and Loja [27].

These figures must be interpreted with caution: They represent only the most severe, symptomatic presentations reaching hospital‐level care, and almost certainly constitute a fraction of the true national burden. In this sense, they are consistent with—but do not directly quantify—possible gaps in screening of blood donors, pregnant women, and at‐risk rural populations, a concern raised in broader Latin American literature [4]. The geographic concentration of severe cases in historically endemic provinces reflects established transmission foci, though comprehensive national Chagas prevalence data from primary surveys remain unavailable for Ecuador. Regarding leishmaniasis, PAHO’s most recent epidemiological report shows that although the overall regional trend presents a slight decrease, cutaneous leishmaniasis has shown increases in countries such as Argentina, Costa Rica, Ecuador, Mexico, and Suriname [5]. The persistence of foci linked to deforestation, agricultural expansion, and occupation of new extractive frontiers reflects the interaction between environmental dynamics and vector exposure, a pattern documented across Latin America [16].

### 3.5. Diseases Captured by the DALY Estimate, Regional Control Strategies, and the Role of WASH

The GBD cause group used in this analysis (Cause 344, “NTDs and malaria”) is an aggregate. Interpreting the DALY comparison therefore requires specifying which diseases it contains, what strategy is used against each elsewhere in Latin America, and where WASH acts on the transmission cycle. Table 5 sets this out for the components relevant to the Region of the Americas. Two features are consequential for interpretation. First, dengue is included within this GBD cause group, which provides a further methodological justification—beyond the conceptual argument given in the Introduction—for analyzing it alongside the classical NTDs. Second, the diseases in the group differ fundamentally in the degree to which WASH is on the causal pathway: It is determinant for STH, schistosomiasis, trachoma, and dengue; indirect and housing‐mediated for Chagas disease; and essentially irrelevant for onchocerciasis, lymphatic filariasis, and malaria. Aggregate DALY rankings therefore cannot be read as an index of WASH performance, nor as a measure of programmatic quality, since a country’s position is driven by whichever component disease dominates its burden.

**TABLE 5:** Component diseases of GBD Cause 344 relevant to Latin America: dominant control strategy outside Ecuador and the role of WASH in elimination.

Disease	Dominant regional strategy (illustrative country)	Role of WASH in control or elimination
Soil‐transmitted helminthiases	School‐based PCT with albendazole or mebendazole, integrated with deworming and nutrition programs (Brazil, Peru, Bolivia) [6]	Determinant. Access to and use of improved sanitation is associated with roughly halved odds of infection (pooled OR 0.51, 95% CI 0.44–0.61), and 0.66 (0.57–0.76) in adjusted analyses; PCT alone does not interrupt transmission [10, 33]
Schistosomiasis	PCT plus snail control and sanitation investment; Brazil is the only country in the Region with substantial residual endemicity [32]	Determinant. Ecological studies across LAC show prevalence tracking sewage and water‐supply coverage; sanitation is the principal driver of the long‐term decline observed in Brazil [34]
Chagas disease	Subregional intergovernmental initiatives (Southern Cone, Andean, Central American) combining residual insecticide spraying, housing improvement, and universal blood‐bank screening; Brazil certified interruption of *Triatoma infestans* transmission in 2006 [7]	Indirect but material. Operates through housing quality and peridomestic hygiene rather than the fecal–oral route; wall plastering, floor and roof replacement remove triatomine refugia [9]
Leishmaniases	Passive case detection with etiological treatment, vector control, and canine reservoir management for visceral disease (Brazil) [5]	Marginal as a direct pathway; peridomestic waste accumulation and substandard housing act as modifiers of vector exposure [16]
Onchocerciasis	Biannual community‐wide MDA with donated ivermectin under OEPA, with entomological and serological verification (Colombia, Guatemala, Mexico) [12, 31]	Negligible. Transmission is riverine and vector‐dependent and is unaffected by household water or sanitation status
Lymphatic filariasis	MDA with albendazole plus diethylcarbamazine, plus morbidity management (Dominican Republic, Guyana, Haiti, Brazil) [6]	Adjunct only, through hygiene‐based limb care to prevent acute dermatolymphangioadenitis
Trachoma	The SAFE strategy—surgery, antibiotics, facial cleanliness, environmental improvement (Brazil, Colombia, Guatemala, Mexico, Peru) [6]	Determinant and explicit: the F and E components of SAFE are WASH interventions
Dengue and other arboviruses	Integrated vector management, entomological surveillance, and novel biological tools such as *Wolbachia* releases (Brazil, Colombia) [16, 18]	Determinant. Intermittent piped supply drives household water storage, generating oviposition sites; solid waste accumulation supplies additional containers [16]
Malaria	Vector control with insecticide‐treated nets and indoor residual spraying, diagnosis, and radical treatment (Brazil, Peru, Colombia	Not a WASH‐mediated disease; included because the GBD cause group aggregates malaria with NTDs

*Note:* PCT = preventive chemotherapy, OEPA = Onchocerciasis Elimination Program for the Americas, LAC = Latin America and the Caribbean, SAFE = surgery, antibiotics, facial cleanliness, environmental improvement. Strategies are those reported for countries of the Region.

Abbreviations: MDA = mass drug administration, OR = odds ratio.

### 3.6. GBD 2023 Data: NTD and Malaria Burden in South America

The Global Burden of Disease Study 2023 provides updated estimates for NTDs and malaria (GBD Cause 344). Table 2 presents DALY rates (both sexes, all ages) for selected South American countries, including Ecuador and its regional comparators.

The rationale for selecting these four comparators is detailed in Section 2.5. Ecuador’s DALY rate of 193.8 per 100,000 (95% UI: 139.4–267.6) is substantially lower than Bolivia (388.9) and Brazil (313.3), but higher than Colombia (149.6). This intermediate position reflects Ecuador’s successful elimination of onchocerciasis while maintaining persistent burdens from Chagas disease, leishmaniasis, and arboviruses. Because cause 344 is an aggregate (Section 3.5), the ordering is driven by different component diseases in each country—Chagas disease in Bolivia, schistosomiasis and dengue in Brazil—and the uncertainty intervals of Ecuador, Colombia, and Peru overlap substantially. The comparison should therefore be read as a description of burden composition rather than as a ranking of programmatic performance.

### 3.7. WASH Coverage in Latin America and Ecuador

The JMP regional summary for LAC documents that in 2020, 75% of the population used safely managed drinking water and 96% had at least basic water services; in sanitation, 34% had access to safely managed services and 89% to basic services, while open defecation was reduced to less than 1% [22]. Despite these achievements, 17 million people still lacked basic water service and 72 million lacked basic sanitation, with strong inequalities between urban and rural areas [22].

In Ecuador, SDG country profiles indicate that the proportion of population using basic drinking water services increased from 82.3% in 2000 to 95.7% in 2022, while the proportion using safely managed sanitation went from 40.0% to 41.6% in the same period [23]. Despite high basic water coverage, progression toward “safely managed” service levels is slow, particularly in sanitation.

The MAATE–UNICEF report documents that rural Ecuador is overrepresented in the use of unimproved water sources (7%) or surface water (6%), and that 8% use limited basic sanitation, 1% use unimproved sanitation services, and approximately 3% still practice open defecation [24]. The study also quantifies significant economic losses associated with morbidity from diarrhea, parasitoses, and related diseases, as well as impacts on productivity and educational outcomes.

### 3.8. Ecuador Compared to LAC: Comparative Indicators

Tables 3 and 4 present selected indicators comparing Ecuador’s performance with the regional average for LAC (WASH data) and the Region of the Americas (disease data).

Tables 3 and 4 illustrate the dual profile of Ecuador’s public health situation. On WASH coverage, Ecuador broadly tracks the regional average for basic drinking water service but lags on the “safely managed” tier, with significant residual rural deficits. On disease burden, the country shares the high dengue burden and persistence of Chagas and leishmaniasis common to the Region, while simultaneously having achieved certified onchocerciasis elimination.

### 3.9. Relationship Between WASH and NTD Burden

Figure 2 illustrates the ecological relationship between safely managed sanitation coverage and DALY rates for NTDs and malaria across five South American countries. Ecuador (highlighted) shows a distinctive profile: with sanitation coverage (41.6%) slightly above the LAC average (34%), its NTD + malaria burden (193.8/100,000) is lower than Bolivia and Brazil but remains substantial. The five countries do not align along a monotonic inverse gradient, and the apparent positive ordering of burden with sanitation coverage is a composition effect rather than a causal signal: Cause 344 aggregates malaria, whose burden in Bolivia, Brazil, and Peru is concentrated in Amazon‐basin transmission foci that are not WASH‐mediated (Table 5). Read with that caveat, the figure aligns with the broader argument that while sanitation improvements contribute to NTD control, they must be accompanied by targeted interventions for diseases such as Chagas and leishmaniasis that have non–fecal–oral transmission routes.

## 4. Discussion

The findings of this narrative review illustrate a double paradox that characterizes Latin American public health, with Ecuador as a particularly instructive case study. On one hand, the Region of the Americas and Ecuador specifically have demonstrated that elimination of historical NTDs is achievable through intensive vertical programs of MDA, associated with strong community engagement and sustained entomological and serological surveillance. Onchocerciasis elimination in Ecuador, certified in 2014, constitutes a model achievement in a hyperendemic focus with a highly efficient vector [19, 20]. As the theoretical literature anticipated, this success was possible precisely because onchocerciasis transmission was primarily vector‐focused, geographically contained, and amenable to long‐term MDA with a highly efficacious drug [7, 8].

On the other hand, the same Region records the largest dengue outbreak in decades, with more than 12.6 million cases in 2024 and millions of additional cases in 2025, in a context of accelerated urbanization, climate change, and structural deficits in urban environmental management [9, 21]. This underscores the limitations of approaches focused solely on MDA or classical vector control, without sustained interventions on housing, waste management, urban services, and community education [14, 16]. It is the author’s interpretation—consistent with established theoretical frameworks [7, 8]—that dengue’s resistance to elimination reflects not a programmatic failure per se, but the structural absence of the environmental and socioeconomic preconditions that made onchocerciasis elimination possible. This distinction is reinforced by comparing the trajectories of different NTD categories.

MDA has driven the elimination of diseases such as onchocerciasis and lymphatic filariasis in specific foci across Latin America, because long‐term MDA can reduce the parasite reservoir below the transmission threshold in settings where the vector is the principal interface [6, 19, 20]. For STH, however, periodic PCT reduces individual worm burden and associated morbidity but cannot interrupt transmission in communities where open defecation, unimproved water sources, and overcrowding persist [8, 14]. This structural constraint explains why the WHO NTD Roadmap 2021–2030 emphasizes WASH integration as a co‐strategy alongside PCT for STH control, with Target 3 explicitly calling for cross‐cutting WASH interventions to support the control and elimination of multiple NTDs including STH, schistosomiasis, trachoma, and lymphatic filariasis [6]. In Ecuador, rural WASH deficits documented by the MAATE–UNICEF report [24] suggest that STH control gains from MDA‐based programs remain vulnerable to reinfection in the absence of complementary infrastructure investment aligned with WHO’s Target 3 framework.

The persistence of Chagas disease and leishmaniasis in Ecuador similarly reflects the interaction between social determinants (poverty, migration, occupation of new agroextractive frontiers) and health systems that maintain gaps in early diagnosis, screening of at‐risk populations, and access to etiological treatment [4, 5]. The 439 severe hospitalized Chagas cases identified in Ecuador between 2013 and 2019 [27] are best understood as a visible fraction of a larger, predominantly asymptomatic or undiagnosed burden—a pattern consistent with global Chagas epidemiology [4, 26]. The epidemiological transition of Chagas disease, with chronic forms increasingly presenting in urban contexts and in nonendemic countries through migration, requires rethinking surveillance and care models beyond the traditional rural vector‐control paradigm.

The role of WASH emerges as a cross‐cutting axis, but the relationship between WASH deficits and NTD persistence operates through distinct, disease‐specific mechanisms that must be disaggregated for effective intervention design [33, 35]. *Water storage practices* create breeding sites for *Aedes aegypti*, the principal dengue vector in urban and periurban Latin America: uncovered household water containers, cisterns, and improvised rainwater receptacles provide the stagnant, clean‐water oviposition sites that drive vector proliferation in settings where piped water service remains intermittent or unavailable [16, 18]. This mechanism explains why dengue persists even in cities with nominally high water‐service coverage: The problem is not lack of infrastructure per se, but the behavioral and infrastructural patterns induced by unreliable supply.

*Open defecation and inadequate sanitation* constitute the primary environmental contamination pathway for STH—*Ascaris lumbricoides*, *Trichuris trichiura*, and hookworm—which complete their transmission cycle through fecal contamination of soil and subsequent oral or percutaneous exposure [33, 36]. A systematic review and meta‐analysis by Strunz et al. documented that access to improved sanitation reduced STH odds by 33%, and that the effect was strongest in contexts where sanitation interventions were accompanied by hygiene promotion and handwashing with soap [33]. In Ecuador, the persistence of 3% open defecation prevalence in rural areas and 8% limited or unimproved sanitation access [24] creates sustained environmental reservoirs for helminth reinfection, even in populations that receive periodic PCT. This explains why STH control through MDA alone—unlike onchocerciasis—cannot achieve elimination without concurrent environmental modification: the treatment–reinfection cycle continues as long as fecal–oral pathways remain open [8].

*Housing materials and construction quality* directly mediate *Trypanosoma cruzi* transmission through domestic infestation by triatomine vectors. Adobe walls, palm‐thatch roofs, and earthen floors provide the cracks, crevices, and refuge microhabitats that triatomines require for daytime resting and nighttime human blood‐feeding [4, 9]. In rural Ecuador, particularly in historically endemic provinces such as Loja, El Oro, and Manabí, substandard housing remains a structural driver of vector‐borne Chagas transmission, though congenital transmission and oral outbreaks have increasingly been documented [27]. Vector control through housing improvement—plastering walls, installing metal roofs, replacing dirt floors—has been shown to reduce domestic triatomine densities more effectively than residual insecticide spraying alone, because structural modifications eliminate the ecological niches that insecticides temporarily suppress but do not destroy [4, 9].

These mechanistic distinctions are not merely taxonomic; they have direct policy implications. Although Latin America shows high coverage of basic water and sanitation services, progress toward safely managed service levels is heterogeneous and insufficient to achieve SDG targets [22, 28]. In Ecuador, high basic water coverage (95.7%) coexists with a large proportion of rural population still exposed to unimproved or surface water (13% combined) and inadequate sanitation (41.6% safely managed), with documented economic impacts from diarrhea, parasitoses, and related morbidity [24]. The link between WASH deficits and NTD persistence is well‐established at the population level [33, 35], though the ecological design of this analysis—and the aggregate secondary data it employs—does not permit causal attribution at the individual level (see Section 4.2). Effective NTD control requires interventions tailored to the specific transmission pathways: intermittent water‐service regularization and vector‐proof storage for dengue, latrine construction, and hygiene promotion for STH, housing improvement for Chagas—not a generic “WASH package” applied uniformly [6].

From a health policy perspective, PAHO’s Communicable Disease Elimination Initiative proposes an integrated framework that goes beyond pathogen‐specific elimination targets, incorporating cross‐cutting action axes such as WASH, social determinants, life course approach, and primary care strengthening [17]. However, effective implementation of this framework requires translating regional commitments into national plans with clear budgets, intersectoral governance mechanisms, and information systems capable of integrating health, environment, and social development data. Concrete operational examples from the Region—including Brazil’s integration of STH PCT with conditional cash transfer programs, and Colombia’s community‐based onchocerciasis surveillance model—illustrate that success is achievable when health programs are embedded within broader social policy frameworks [7, 25].

Yet, infrastructure alone does not guarantee behavior change or sustained health outcomes. The social and behavioral science literature on WASH and NTD control identifies critical noninfrastructural determinants that mediate intervention effectiveness. The integrated behavioral model for WASH (IBM‐WASH), developed by Dreibelbis et al., proposes that WASH behavior change is shaped by contextual factors (physical environment, social norms), psychosocial factors (knowledge, attitudes, self‐efficacy), and habitual dimensions (routine, convenience) [37, 38]. In rural Ecuador, the persistence of open defecation despite latrine availability in some communities reflects not solely infrastructure gaps but also normative beliefs about appropriate defecation sites, perceived cleanliness of latrines, and competing domestic priorities [39]. Community‐led total sanitation (CLTS)—a participatory approach that triggers collective behavior change through community‐facilitated recognition of fecal contamination pathways—has demonstrated effectiveness in reducing open defecation in comparable Latin American contexts, though evidence specific to Ecuador remains limited [39].

For Chagas disease, the behavioral and social determinants extend beyond vector‐control infrastructure. Many chronic Chagas patients in Ecuador remain undiagnosed because of stigma, lack of awareness of infection risk, geographic barriers to diagnostic facilities, and distrust of health systems rooted in historical marginalization of rural and Indigenous populations [40, 41]. Unlike acute infectious diseases where symptomatic presentation drives health‐seeking behavior, chronic Chagas infection is predominantly asymptomatic for decades, creating a behavioral paradox: The individuals most in need of etiological treatment are precisely those least likely to seek it [9, 40]. Screening programs targeting blood donors, pregnant women, and residents of endemic provinces have been shown to increase case detection, but sustained success depends on community education about transmission routes, reduction of treatment‐related stigma, and provision of decentralized diagnostic and treatment services [4, 41].

The onchocerciasis elimination success in Ecuador’s Esmeraldas focus offers instructive lessons about the behavioral determinants of programmatic effectiveness. Community participation was not an incidental feature but a core operational strategy: Local health promoters, themselves residents of endemic communities, were trained to distribute ivermectin, document treatment coverage, and educate neighbors about the disease’s etiology and prevention [19]. High MDA coverage (¿85%) was achieved and sustained because the program built trust through culturally appropriate communication, employed community members as program implementers, and maintained visible, consistent presence over two decades [19, 20]. This model contrasts sharply with top‐down interventions that deliver services without engaging local knowledge or building local capacity—a pattern documented as a common failure mode in NTD control programs elsewhere in the Region [14, 25]. Replicating this success for dengue, Chagas, or STH control requires analogous community engagement strategies tailored to each disease’s transmission ecology and the specific behavioral determinants that govern exposure and treatment‐seeking in affected populations.

In Ecuador’s case, integrating accumulated experience in onchocerciasis elimination with the dengue, Chagas, leishmaniasis, and helminthiasis control agendas implies: (a) leveraging community capacities and surveillance networks developed in the Esmeraldas focus to strengthen vector monitoring for other diseases; (b) aligning rural water and sanitation investments with NTD elimination goals and chronic child malnutrition reduction targets; and (c) consolidating integrated surveillance that combines clinical, entomological, environmental, and social information. The contribution of this review is to illustrate that these policy articulations are not hypothetical but grounded in the documented paradox that Ecuador’s own experience embodies.

### 4.1. What the Paradox Consists of, and What It Does Not

The framework set out in the Introduction can now be evaluated against the evidence assembled here. The Ecuadorian contrast is not adequately explained by the geographical confinement of the Esmeraldas focus or by the clinical visibility of river blindness. Focal, visible diseases have persisted for decades elsewhere in the Region in the absence of a delivery architecture, and cutaneous leishmaniasis—also focal and visibly disfiguring—is increasing in Ecuador [5]. What distinguished onchocerciasis was a configuration of four programmatic conditions that acted jointly: a dedicated regional partnership with predictable external financing over more than two decades; an indefinite donation of a single, safe, single‐dose medicine; an intervention simple enough to be delivered by trained community members; and an internationally codified endpoint with explicit criteria for stopping MDA and verifying interruption of transmission [11–13]. The fourth condition deserves emphasis because it is frequently overlooked: A verifiable finish line converts an open‐ended expenditure into a bounded, auditable, politically rewarding commitment, and it is precisely what Chagas disease, STH, leishmaniasis, and dengue lack.

The biological and ecological differences summarized in Table 1 are real and impose hard constraints—sylvatic *T. cruzi* reservoirs, environmentally persistent helminth eggs, and the domestic breeding ecology of *Aedes aegypti* preclude elimination by chemotherapy alone [9, 10, 16]. They are, however, necessary rather than sufficient conditions for the observed divergence: They explain why the onchocerciasis *tool* is not transferable, but not why the corresponding *investment* in housing, sanitation, and water‐supply continuity has not materialized. That second question is institutional and political–economic. The decisive levers sit in ministries of housing, water, and municipal government, are financed domestically, yield benefits over electoral time horizons longer than a single administration, and cannot be certified by an international verification team [8, 14, 25].

The practical consequence is a specific and testable claim about transferability. What Ecuador can carry forward from Esmeraldas is not the vertical model but its human infrastructure: trained community distributors, functioning entomological surveillance, and an established relationship of trust with historically marginalized Afro‐Ecuadorian and Indigenous populations [19]. What cannot be carried forward is the financing‐and‐endpoint architecture, which must be substituted domestically—through budgeted intersectoral targets, WASH investment tied to NTD indicators, and nationally defined milestones that reproduce the disciplining function of a verification standard. Stated in this form, the paradox is a policy diagnosis rather than an observation about irony: It identifies which component of the success was contingent on external conditions and therefore must be rebuilt with domestic instruments.

### 4.2. Limitations

Several methodological limitations must be explicitly acknowledged.

*Ecological inference.* This study compares aggregate indicators at the national and regional level. Such ecological analyses are inherently susceptible to the ecological fallacy: Associations between aggregate‐level variables (e.g., provincial WASH coverage and NTD incidence) cannot be assumed to reflect individual‐level relationships [42]. Aggregate data mask substantial within‐group heterogeneity, particularly among marginalized communities—such as Indigenous populations in the Ecuadorian Amazon or Afro‐descendant communities in Esmeraldas—where disease burdens and WASH access may differ dramatically from national averages.

*Surveillance heterogeneity and underreporting.* Countries and subnational regions in Latin America differ substantially in their case‐detection capacity, reporting periodicity, and diagnostic infrastructure. NTDs with long asymptomatic phases—including Chagas disease, lymphatic filariasis, and leishmaniasis—are systematically underreported in passive surveillance systems [43]. Published PAHO and WHO figures should therefore be interpreted as minimum confirmed estimates rather than true prevalence. In some remote localities, the reality of NTD burden and environmental conditions may be substantially worse than what official records reflect.

*Temporal and definitional inconsistencies.* The indicators presented in Tables 2, 3, and 4 originate from different reporting years (2013–2025), different geographic denominators (LAC vs. Region of the Americas), and different definitional standards (e.g., “basic” vs. “safely managed” services under the JMP ladder framework). While year labels and source annotations are attached to each indicator, cross‐indicator comparisons must be interpreted with caution and should not be treated as strictly contemporaneous or methodologically homogeneous.

*Reliance on secondary data.* The absence of primary field surveys means that key claims rest on institutional reports and peer‐reviewed publications that themselves draw on surveillance systems with variable completeness. A nationally representative parasitological survey for intestinal helminths and other NTDs in Ecuador—currently absent in the published literature—would substantially strengthen the evidence base for future policy analyses and is recommended as a national priority.

*Aggregation of the GBD cause group.* Cause 344 aggregates diseases with heterogeneous transmission routes, strategies, and WASH dependence (Table 5). Country‐level differences in the aggregate rate reflect burden composition rather than comparative program effectiveness, and the overlapping uncertainty intervals of Ecuador, Colombia, and Peru preclude ordinal interpretation. Disease‐specific DALY decomposition, which the GBD Results Tool supports but which falls outside the scope of this synthesis, would be required to attribute burden differences to specific programs.

*Scope of the comparative framework.* The analysis compares Ecuador against LAC or the Region of the Americas as composite units. Within the Region, there is substantial heterogeneity: Brazil alone accounts for the majority of dengue burden, and between‐country variation in WASH and NTD indicators is large. Inferences about “the Region” must be understood as aggregate tendencies rather than uniform conditions.

*Visual presentation.* Figure 2 presents an ecological comparison of sanitation coverage and NTD burden across five South American countries. The overlapping confidence intervals and varied population sizes create visual interpretation challenges, particularly for Brazil, Peru, and Bolivia. The figure is therefore presented as a purely descriptive scatter plot with uncertainty intervals and without any fitted trend line, and should be read as an illustration of the joint distribution of the two indicators rather than as evidence of a dose–response relationship.

## 5. Conclusions

The Ecuadorian experience confirms that targeted elimination of NTDs such as onchocerciasis is feasible through intensive, sustained interventions—specifically MDA combined with rigorous entomological surveillance, community participation, and international programmatic support. The divergence between this achievement and the persistence of other NTDs is best understood as institutional and programmatic rather than merely biological: onchocerciasis benefited from a dedicated externally financed initiative, a donated single‐dose medicine, an intervention simple enough for community delivery, and a verifiable elimination endpoint, none of which is available for Chagas disease, STH, leishmaniasis, or dengue. Ecuador’s 2014 certification of onchocerciasis elimination stands as a reference achievement for the Region and demonstrates the potential of well‐resourced, long‐term vertical programs. This analysis, framed explicitly as an integrative policy synthesis rather than a primary analytic study, documents how this success coexists with the persistence of other NTDs and arboviruses that MDA alone cannot resolve.

Latin America presents high coverage levels of basic WASH services, but progress toward safely managed service levels remains insufficient and marked by deep urban–rural disparities. In Ecuador, rural WASH deficits translate into significant health and economic costs, and constitute a structural driver of persistence for helminthiases, intestinal parasitoses, and other environmentally transmitted diseases. These gaps are particularly acute in remote and historically marginalized communities, where aggregate national figures obscure substantially worse local conditions. Addressing these deficits is not only a public health imperative but a prerequisite for sustaining the NTD elimination gains already achieved.

PAHO’s Communicable Disease Elimination Initiative offers a promising framework to articulate specific NTD elimination goals with water and sanitation, housing, environment, and inequality‐reduction policies. Translating this framework into national plans requires sustained financial commitments, intersectoral governance mechanisms, and primary healthcare systems oriented toward equity and territorial reach. A national parasitological and NTD surveillance survey for Ecuador would be a concrete and high‐value contribution toward closing the evidence gap identified in this review. Ultimately, the transformation of punctual elimination achievements into structural health equity gains demands political commitment to address the conditions of poverty and environmental precariousness that have historically defined neglect in the first place.

## Funding

No funding was received for this manuscript.

## Conflicts of Interest

The author declares no conflicts of interest.

## Supporting Information

Additional supporting information can be found online in the Supporting Information section.

## Supporting information


**Supporting Information 1** Supporting File S1 (Supporting_File_S1.docx). Complete data extraction from the Institute for Health Metrics and Evaluation Global Burden of Disease Results Tool for GBD Cause 344 (neglected tropical diseases and malaria), both sexes, all ages, reference Year 2021, for Bolivia, Brazil, Colombia, Ecuador, and Peru. The file states the full query specification and reproduces, in Table S1, every record returned by the Results Tool: the point estimate and the 95% uncertainty interval for each of the three metrics released by the tool (number of DALYs, percentage of all‐cause DALYs, and rate per 100,000 population), together with the data citation generated at download. It is the primary source of the estimates reported in Table 2 and plotted in Figure 2 and is provided so that the burden comparison can be reproduced without re‐querying the Results Tool.

**Supporting Information 2** PRISMA 2020 CHECKLIST.

## Data Availability

The data that support the findings of this study are derived from the following resources available in the public domain: Pan American Health Organization (PAHO) reports and databases (https://www.paho.org), World Health Organization (WHO) documents and data repositories (https://www.who.int/data), the WHO/UNICEF Joint Monitoring Programme for Water Supply, Sanitation and Hygiene (JMP) (https://washdata.org), statistical data from Ecuador’s Ministry of Public Health (https://www.salud.gob.ec), United Nations Sustainable Development Goals (SDG) country profiles (https://unstats.un.org/sdgs/dataportal), World Bank Development Indicators (https://data.worldbank.org), and peer‐reviewed scientific publications as detailed in the References section.
