# Supplementary material for: Paradoxes of Elimination and Persistence: Neglected Tropical Diseases in Ecuador Compared to Latin America and the Caribbean
Source: J Trop Med. 2026 Sep 20;2026:2977701. doi: 10.1155/jotm/2977701 (PMC13591011; doi:10.1155/jotm/2977701)
Supplement: Supplementary file 1 [file JOTM-2026-2977701-s001.docx]

**PRISMA 2020 CHECKLIST**

for Narrative Reviews and Systematic-Descriptive Comparisons

NTDs Ecuador Manuscript

# COMPLIANCE SUMMARY

**Este checklist evalua el manuscrito contra 32 ItElms PRISMA 2020 aplicables a revisiones narrativas sistematizadas.**

Escala: Y = SÍ (cumple), P = PARCIALMENTE (cumple con restricciones), N = NO/N.A.

| **Status** | **YES (Y)** | **PARTIAL (P)** | **NO/N.A. (N)** |
| --- | --- | --- | --- |
| Applicable Items | **20** | **6** | **6** |
| Percentage | **58%** | **17%** | **17%** |
| **COMBINED COMPLIANCE** | **75% (26 of 32 applicable items)** | | |

# CRITICAL FINDINGS

- **CUMPLE:** 20/32 items (58%) - Búsqueda sistematizada, criterios de inclusión, métodos documentados, datos actualizados GBD 2023
- **PARCIAL:** 6/32 items (17%) - Sin formal GRADE rating, sin diagrama PRISMA flow, datos faltantes no cuantificados
- **N/A:** 6/32 items (17%) - Protocolo preregistrado no aplicable, riesgo de sesgo no evaluado formalmente

# ITEMS REQUIRING ACTION

1. **ADD PRISMA FLOW DIAGRAM (CRITICAL)**

Manuscrito carece de diagrama visual mostrando: registros identificados > screening > elegibilidad > inclusion

2. **QUANTIFY DOCUMENT FLOW IN METHODS (IMPORTANT)**

Agregar en Section 2.2: numero de registros identificados en cada base de datos, cuantos fueron excluidos en screening, cuantos en full-text review

3. **CONSIDER GRADE RATING (RECOMMENDED)**

Aplicar GRADE (Alta/Moderada/Baja/Muy Baja) a indicadores principales para evaluacion de certeza de evidencia

# DETAILED ITEM ASSESSMENT

| **#** | **Item** | **Y/P/N** | **Section** | **Note** |
| --- | --- | --- | --- | --- |
| 1 | Title | **Y** | P. 1 | Clear, identifies as narrative review of secondary data |
| 2a | Abstract: Objective | **Y** | P. 1 | Explicit objective stated |
| 2b | Abstract: Eligibility | **P** | Methods | Detailed in Meth 2.2, condensed in abstract |
| 2c | Abstract: Sources | **Y** | P. 1 | PAHO, WHO, JMP listed |
| 3a | Intro: Context | **Y** | P. 1-3 | Hotez et al., Engels & Zhou cited |
| 3b | Intro: Objectives | **Y** | P. 3 | Policy synthesis framing explicit |
| 5 | Information Sources | **Y** | P. 3-4 | Databases, repositories, 2015-2026 |
| 6a | Search Strategy | **Y** | P. 3-4 | Terms documented |
| 7 | Study Selection | **Y** | P. 4 | Inclusion criteria explicit |
| 8a | Data Extraction | **Y** | P. 4 | Variables listed in 2.3 |
| 9a | PRISMA Flow Diagram | **N** | MISSING | CRITICAL: Add search flowchart |
| 10a | Tables/Characteristics | **Y** | P. 6-8 | Tables 1-3 with source notes |
| 11a | Bias Assessment | **P** | P. 12 | Limitations section but no GRADE |
| 12a | Synthesis Methods | **Y** | P. 5 | Comparative descriptive analysis |
| 13a | Results: Quantitative | **Y** | P. 6-8 | DALY rates, dengue cases, WASH % |
| 15a | Discussion Context | **Y** | P. 9-11 | Integrated with theory/policy |
| 15b | Limitations Discussion | **Y** | P. 12-13 | Comprehensive 350-word section |
| 18b | Data Availability | **Y** | P. 13-14 | All public sources listed |

**Conclusion**

**El manuscrito cumple con 75% de criterios PRISMA aplicables. Con adicion de diagrama de flujo, cuantificacion de documentos, y GRADE rating, seria 100% PRISMA-compliant.**
