# Supplementary material for: Paradoxes of Elimination and Persistence: Neglected Tropical Diseases in Ecuador Compared to Latin America and the Caribbean
Source: J Trop Med. 2026 Sep 20;2026:2977701. doi: 10.1155/jotm/2977701 (PMC13591011; doi:10.1155/jotm/2977701)
Supplement: Supplementary file 2 [file JOTM-2026-2977701-s002.docx]

**Supplementary File S1**

*Paradoxes of elimination and persistence: neglected tropical diseases in Ecuador compared to Latin America and the Caribbean*

Manuscript ID 2977701 — Journal of Tropical Medicine

**S1.1 Contents**

This file contains the complete Global Burden of Disease extraction underlying the burden comparison reported in the manuscript. Table S1 reproduces, without modification, every record returned by the Institute for Health Metrics and Evaluation (IHME) Global Burden of Disease Results Tool for the query specified in Section S1.2. It is the source of the estimates reported in Table 3 and plotted in Figure 2 of the manuscript, and is provided so that the comparison can be reproduced without re-querying the Results Tool.

**S1.2 Query parameters**

| **Data source** | IHME Global Burden of Disease Study 2023 (GBD 2023) Results Tool, https://vizhub.healthdata.org/gbd-results/ |
| --- | --- |
| **Measure** | DALYs (disability-adjusted life years) |
| **Cause** | Cause 344 — Neglected tropical diseases and malaria |
| **Locations** | Bolivia, Brazil, Colombia, Ecuador, Peru |
| **Sex** | Both |
| **Age** | All ages (not age-standardised) |
| **Metrics** | Number; percentage of all-cause DALYs; rate per 100,000 population |
| **Reference year** | 2021 |
| **Uncertainty** | 95% uncertainty intervals as released by the Results Tool |
| **Extraction reference** | IHME-GBD_2023_DATA-f2def522-1 |

**Table S1. Complete GBD 2023 extraction for cause 344 (neglected tropical diseases and malaria), both sexes, all ages, 2021, with 95% uncertainty intervals.**

| **Country** | **Metric** | **Estimate** | **Lower 95% UI** | **Upper 95% UI** |
| --- | --- | --- | --- | --- |
| **Bolivia** | Number of DALYs | 45,855 | 33,834 | 61,604 |
|  | Percentage of all-cause DALYs (%) | 0.845 | 0.653 | 1.082 |
|  | Rate per 100,000 population | 388.9 | 286.9 | 522.4 |
| **Brazil** | Number of DALYs | 660,626 | 499,664 | 874,580 |
|  | Percentage of all-cause DALYs (%) | 0.826 | 0.668 | 1.038 |
|  | Rate per 100,000 population | 313.3 | 237.0 | 414.8 |
| **Colombia** | Number of DALYs | 76,480 | 59,884 | 99,420 |
|  | Percentage of all-cause DALYs (%) | 0.470 | 0.394 | 0.557 |
|  | Rate per 100,000 population | 149.6 | 117.1 | 194.5 |
| **Ecuador** | Number of DALYs | 33,842 | 24,344 | 46,733 |
|  | Percentage of all-cause DALYs (%) | 0.637 | 0.483 | 0.816 |
|  | Rate per 100,000 population | 193.8 | 139.4 | 267.6 |
| **Peru** | Number of DALYs | 99,842 | 69,208 | 140,377 |
|  | Percentage of all-cause DALYs (%) | 0.759 | 0.559 | 0.993 |
|  | Rate per 100,000 population | 281.8 | 195.3 | 396.2 |

DALY: disability-adjusted life year; UI: uncertainty interval. Percentages are expressed as the share of all-cause DALYs. The rate per 100,000 population is the metric reported in Table 3 and plotted in Figure 2 of the manuscript; it is a crude all-ages rate, not an age-standardised rate.

**S1.3 Data citation**

Global Burden of Disease Collaborative Network. Global Burden of Disease Study 2023 (GBD 2023) Results. Seattle, United States: Institute for Health Metrics and Evaluation (IHME), 2024. Available from https://vizhub.healthdata.org/gbd-results/

This citation is reproduced verbatim from the citation file generated by the Results Tool at the time of download.

**S1.4 Reuse**

GBD results are made available by IHME under the Free-of-Charge Non-commercial User Agreement. The extraction is reproduced here for verification and reproducibility of the analysis reported in the manuscript.
